# Apigenin role as cell-signaling pathways modulator: implications in cancer prevention and treatment

**DOI:** 10.1186/s12935-021-01888-x

**Published:** 2021-04-01

**Authors:** Zeeshan Javed, Haleema Sadia, Muhammad Javed Iqbal, Shazia Shamas, Kausar Malik, Rais Ahmed, Shahid Raza, Monica Butnariu, Natalia Cruz-Martins, Javad Sharifi-Rad

**Affiliations:** 1 Office for Research Innovation and Commercialization, Lahore Garrison University, Sector-C, Phase VI, DHA, Lahore, 54792 Pakistan; 2 Department of Biotechnology, Engineering and Management Sciences, Balochistan University of Information Technology, Quetta, 87100 Pakistan; 3 Department of Biotechnology, Faculty of Sciences, University of Sialkot, Sialkot, Pakistan; 4 Department of Zoology, University of Gujrat, Gujrat, Pakistan; 5 Center for Excellence in Molecular Biology, University of the Punjab, Lahore, Pakistan; 6 Department of Microbiology, Cholistan University of Veterinary and Animal Sciences, Bahawalpur, Pakistan; 7 Banat’s University of Agricultural Sciences and Veterinary Medicine “King Michael I of Romania” From Timisoara, Calea Aradului 119, 300645 Timis, Romania; 8 Faculty of Medicine, University of Porto, Alameda Prof. Hern.Ni Monteiro, 4200-319 Porto, Portugal; 9 Institute for Research and Innovation in Health (i3S), University of Porto, 4200-135 Porto, Portugal; 10 Laboratory of Neuropsychophysiology, Faculty of Psychology and Education Sciences, University of Porto, 4200-135 Porto, Portugal; 11 Phytochemistry Research Center, Shahid Beheshti University of Medical Sciences, Tehran, Iran; 12 Facultad de Medicina, Universidad del Azuay, Cuenca, Ecuador

**Keywords:** Apigenin, MicroRNAs, Cell signaling, Nano-formulations, Therapeutic benefits

## Abstract

Cancer is a complex disease orchestrated by various extrinsic and intrinsic pathways. In recent years, there has been a keen interest towards the development of natural extracts-based cancer therapeutics with minimum adverse effects. In pursuit of effective strategy, a wide variety of natural products-derived compounds have been addressed for their anticancer effects. Apigenin is a naturally-occurring flavonoid present abundantly in various fruits and vegetables. Decades of research have delineated the pharmacological and biological properties of apigenin. Specifically, the apigenin-mediated anticancer activities have been documented in various types of cancer, but the generalized scientific evidence encompassing various molecular interactions and processes, such as regulation of the apoptotic machinery, aberrant cell signaling and oncogenic protein network have not been comprehensively covered. In this sense, in this review we have attempted to focus on the apigenin-mediated regulation of oncogenic pathways in various cancers. We have also addressed the cutting-edge research which has unveiled the remarkable abilities of apigenin to interact with microRNAs to modulate key cellular processes, with special emphasis on the nano-formulations of apigenin that can help their targeted delivery and can be a therapeutic solution for the treatment of various cancers.

## Introduction

Cancer is a multifactorial disease with an intricate cell landscape featured by a set of complex molecular interactions and mechanisms [1]. The use of high throughput technologies has enabled to unveil the near to complete resolution of tumor microenvironment. Among them, genomic and proteomic-based approaches have delineated over the years the molecular oncology of tumor cells [2]. With such advances, it has become possible to understand the key mechanisms (gene suppression, overexpression, mutations in the genetic framework at epigenetic/genetic levels, altered cellular signaling pathways and genomic instability) involved in tumor progression, with new data being continuously added every day [3]. Apoptosis is a crucial step for retaining and maintaining a balanced cell growth and altered cell signaling can disturb the apoptosis balance, leading to tumor proliferation and invasiveness [4]. Death receptor (DDR) pathway is initiated by the binding of the tumor necrosis factor (TNF)-related apoptosis inducing ligand (TRAIL) and Fas ligand (FasL) to the transmembrane receptor [5]. This binding ultimately leads to the activation of caspases, such as caspase-8 and caspase-3, that in turn promotes the activation of either extrinsic or intrinsic apoptosis pathways [5]. In addition, it has been reported that an apoptosome is formed by the combination of cytochrome-C, apoptotic protease activating factor (APAF) and caspase-9. This apoptosome triggers the activation of caspase-3, resulting in cell death [6]. Accumulating evidences have suggested that apigenin has ability to modulate the expression of key signaling pathways involved in cancer progression [7–10]. Therefore, it can induce apoptosis in cancer cells. Apigenin is a naturally-occurring flavonoid abundant in fruits, vegetables, herbs and plant-based beverages [11]. Studies suggested that Apigenin has the potential to suppress multiple human cancers including prostate cancer, lung cancer, breast cancer, colorectal cancer, liver cancer, melanoma, glioblastoma, osteosarcoma, leukemia, ovarian cancer, pancreatic cancer and cervical cancer [12, 13] by knockdown cell migration, by activating cell apoptosis and enhancing immunity of cells, through this motility rate of cancer cells decreases [14]. In Colorectal cancer Apigenin blocked the Wnt/β-catenin signaling pathway, silenced the cyclin B1, Cdc2 and Cdc25c expression level, lowered the phosphorylation of Src, Akt and FAK pathway, down regulated the expression level of MMP-9 while up-regulated TAGLN level [12, 15]. In breast cancer, it is observed to decreased the p-JAK1, p-JAK2 and p-STAT3 pathway expression, knockdown cyclin A and B, and CDK1 expression, stimulated the expression level of p21^WAF1/CIP1^, cleaved caspase-8 and -3 and PARP cleavage, activated the acetylation of histone H3, prohibited FN-γ-coupled PD-L1 and STAT1 expression level [16, 17]. In lung cancer, it not only suppressed GLUT1expresssion but also reduced the PI3K/Akt, NF-ĸB/p65 signaling pathway [18, 19]. In case of prostate cancer, apoptosis and reduction in cell proliferation results in the deactivation of IKKα, XIAP, c-IAP1 and c-IAP2, vimentin, survivin, and snail, lowered the D1 and D2, E, Bcl-XL, Bcl-2 expression level, enhanced Bax protein, E-cadherin, p21 and p27, completely blocked the expression of three pathways including Src/FAK/, mad2/3 and Akt [20, 21]**.** In Melanoma cases, it has been reported that apigenin activates the cleaved caspase-3, PARP expression sites, downregulate Twist1, MMP-2/9, VEGF, p-mTOR, ERK1/2 proteins and p-AKT, deactivate FAK/ERK1/2 pathways and silenced STAT3 phosphorylation [22, 23]. In case of leukemia, it induced the caspase-9 and -3 cleavage sites and JNK pathway, blocked JAK/STAT pathway and AkT pathway [24]. In ovarian cancer, it deactivated the FAK and Gli1 pathway’s expression, inhibited CK2α expression level. In Glioblastoma, it lowered TGF-b1 and inhibited the c-Met signaling pathway’s activation [25]. In case of renal cell carcinoma it regulated the expression of ATM signaling pathway. It silenced the GLUT-1 expression in adenoid cystic carcinoma [26]**.** In case of pancreatic cancer, it stabilized the expression of Ikaros. In osteosarcoma, it deactivated Wnt/β-catenin signaling pathway. In cervical cancer, it reduced CK2α expression level [27]. In this sense, the present review aims to focus on the interaction of apigenin with key signaling pathways that can be targeted for development of efficient therapeutics for cancer, on the interactions of apigenin with different non-coding RNAs and even a special focus is given to the current nano-formulations of apigenin that can be used for triggering apoptosis in cancer.

## Interactions of apigenin with different molecular pathways

**Fig. 1:**
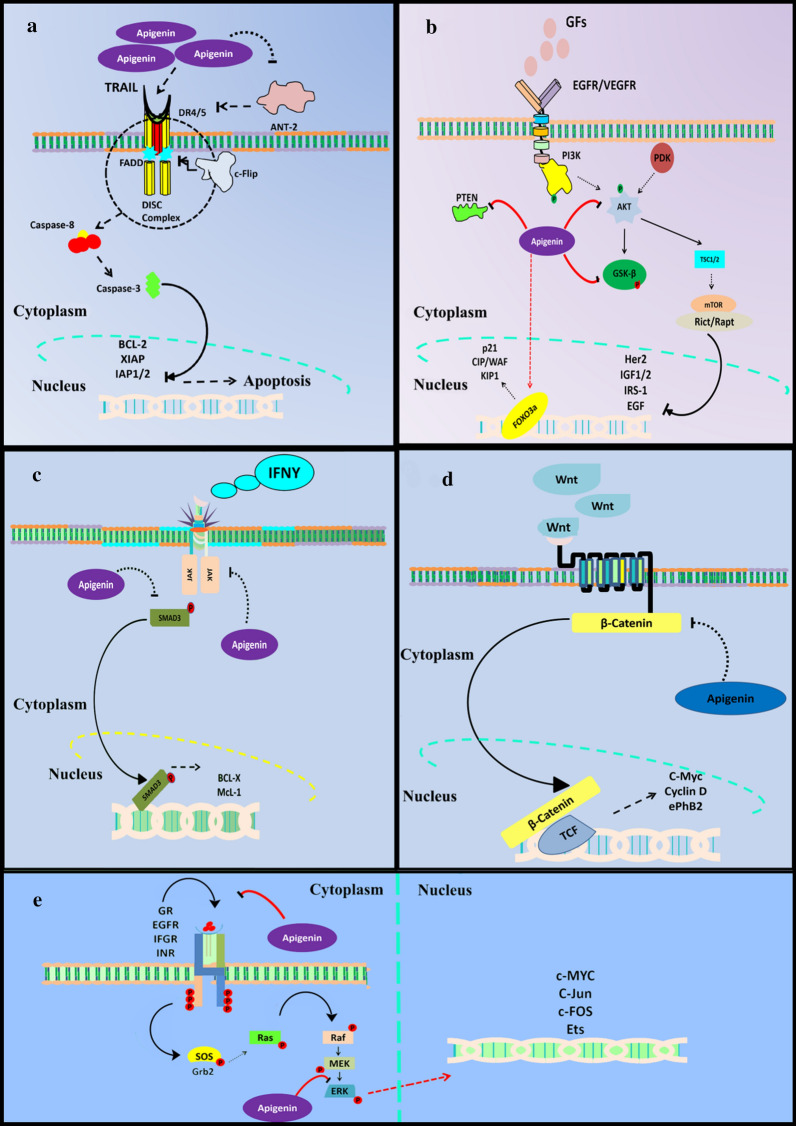
Modulation of various signaling cascades by Apigenin. **a** Apigenin directly influences TRAIL signaling. Apigenin prevents ANT-2 mediated deactivation of DISC complex and thus prevents the activation anti-apoptotic proteins such as the XIAP, BCL-2 and IAP 1/2. **b** Apigenin prevents the phosphorylation of AkT, PTEN, GSKβ and PI3K and prevents downstream activation of transcription factors FOXO3a and thus promotes expression of pro-apoptotic proteins such p21, KIP1 and WAF. **c** Apigenin effects the JAK-STAT signaling through prevention of phosphorylation of JAK and SMAD3 and thus triggers apoptosis. **d** Apigenin mediated modulation of Wnt/β-catenin involves suppression of β-catenin and its downstream translocation to the nucleus. Inhibition of the β-catenin results in the suppression of the target genes involved in proliferation and cell growth. **e** Apigenin attenuates MAPK signaling through regulation of the expression of ERK and upstream binding of various growth factors. The MAPK signaling activation triggers phosphorylation of SOS and Grb2 which in turn promotes Raf/Ras phosphorylation. Apigenin interferes at ERK level and suppress its expression consequently inhibiting the expression of proto-oncogenes such as the c-Myc, c-Jun and c-FOS respectively

The rapid ability of cancer cells to acquire drug resistance against current therapeutic approaches is a major stumbling block for molecular biologists and pharmacologists [28]. As a consequence, it has led scientists to the development of drugs with high specificity and limited cytotoxicity [29]. In the following subsection the apigenin interactions with different molecular pathways is presented and briefly pictured in Fig. 1.

### TRAIL pathway modulation by Apigenin

Mutations in the TRAIL signaling pathway confers drug resistance and reduces apoptosis in various tumors [30]. In fact, TRAIL driven signaling holds many opportunities and strategies to the development of suitable therapeutics that can trigger apoptosis in TRAIL-resistance tumors [31]. Investigations have revealed possible pro-apoptotic and anti-apoptotic proteins that regulate the TRAIL pathway and could be implemented as possible drug intervention strategies that can help in targeting anti-apoptotic proteins [32]. The TRAIL signaling can be activated by various factors, but researchers have delineated 2 mechanisms which are triggered in TRAIL-treated cancer cells [33]. The formation of death inducible signaling complex (DISC) is part of extrinsic mechanism. Indeed, DISC activation is necessary for caspase-8 activation, which in turn triggers the activation of down-stream target, such as caspase-3 [34]. Several studies have disentangled the role of apigenin as TRAIL pathway modulator [35–37]. It has been evidenced in Hep3B cells that apigenin administration reduces the expression of B-cell chronic lymphoma 2 (BCL2), X-linked inhibitor of apoptosis (XIAP), inhibitor of apoptosis protein-1 (IAP-1) and IAP-2, while triggers apoptosis [38]. In anaplastic thyroid carcinoma cells, the combination of apigenin and TRAIL resulted in a decreased BCL2 and increased ERK1 and ERK2 expression [39]. It has been reported that adenine nucleotide translocase-2 (ANT-2) is a negative modulator of TRAIL and can prevent its activation [40]. However, ANT-2 inhibition increases the expression of death receptor (DR5) [40], but apigenin has shown to be able to inhibit the expression of ANT-2 by direct binding to it while initiate apoptosis in colon cancer HCT-116 cells. Also, apigenin administration increased the DR5 and CHOP expression in HCT-116 cells. In addition, apigenin expression also triggered the expression of BID/BAX, cytochrome C, caspase-3, caspase-8 and caspase-9 in HCT-116 cells [41]. In TRAIL-resistant cancer cell lines, such as A549 cell line, apigenin modulated the expression of nuclear factor kappa-B (NFκB), responsible for the activation of several anti-apoptotic proteins. Nonetheless, when used in combination, TRAIL and apigenin, resulted in a persistent accumulation of p65 that negated the proliferative potential of NFκB in A549 cells [42]. In addition to this it has also been reported that apigenin triggers the activation of IκBα, which prevented the A549 cells proliferation [43]. Despite these brimming evidences, still plenty of research is required to disentangle the complexity of TRAIL pathway mediators and modulators in context with apigenin as potential drug target for TRAIL resistant cancer cells. Moreover, microRNAs (miRNAs) have been established as master regulators of plethora of cell processes [44], so that identifying their role in conjunction with apigenin is an interesting new avenue to explore the complex web of TRAIL-miRNA-apigenin interactions.

### PI3K/Akt/mTOR pathway modulation by Apigenin

Gene expression, transcription, translation, proliferation, cell growth and development are the key functions regulated by the PI3K/Akt/mTOR pathway [45]. This signaling cascade has been extensively studied in various types of cancer. Abrogation in PI3K/Akt/mTOR signaling pathway triggers tumor growth, metastasis and invasion [46]. PI3K and Akt are the two essential components of this cellular cascade [47]. Briefly, PI3K is located downstream of the growth factor receptor tyrosine kinase, so that mutations in the Akt protein can consequently disturb downstream signaling and effector protein, ultimately promoting tumor growth and development [48]. Apigenin has been reported to exert its influence on PI3K/AkT/mTOR pathway by modulating the expression of key proteins, such as PTEN, AkT, ERK and others [7]. It has come to light less lately that apigenin inhibits the cross talk between PI3K and AkT via blocking the activity of PI3K. Also, apigenin is able to prevent the AkT phosphorylation via disturbing the ATP binding domain of PI3K and activation of mTORC2 complex [49]. The mTORC2 complex phosphorylates AKT at serine473 [50]. Moreover, it has been reported that mTORC1 and mTORC2 showed varied sensitivity towards rapamycin. Rapamycin destabilizes the both complex while PA stabilizes both complexes [51, 52]. Apigenin also modulates the expression of other molecules, such as the Glycogen synthase kinase-3 (GSK-3). Indeed, the GSK-3β activity has been reported to be regulated by the apigenin, through preventing the GSK-3β phosphorylation by inhibiting the expression of PI3K [9]. Thus, since GSK-3β is a direct target of AkT, apigenin prevents its phosphorylation through PI3K [9].

In addition to this, apigenin has been reported to enhance the expression of tumor suppressor protein, such as the FOXO3a, which in turn enhances the expression of tumor suppressor proteins such as the p21, CIP/WAF and KIP1 [53]. This overexpression of tumor suppressor proteins promotes growth arrest in the tumor cells. Apigenin has also been involved in regulation of the mTOR activity via modulation of the TSC2 expression [54]. Apigenin mediated activation of calcium/calmodulin protein kinase β promotes the activation AMPK which in turn phosphorylates the TSC2 and thus inhibits the mTOR activity [55]. AMPK is a modulator of mTOR pathway and it regulates the mTOR signaling in a feedback loop manner under the expression of phospholipase D (PLD). PLD has been reported to be a downstream signaling target of GTPase Rheb whose activity is being controlled by the TSC complex under the impression of AMPK [56]. From these findings it was concluded that there is a reciprocal feedback mechanism that involves AMPK, PLD and mTOR in cancer cells and can be targeted for devising new therapeutics. In addition, AMPK has been reported to inhibit the raptor protein, a necessary component of mTORC1 [57]. This inhibition prevents the mTOR activity. Altogether, these studies underline that apigenin directly inhibits the expression of AKT and thus prevents tumor cells proliferation in different cancers.

### JAK/STAT signaling modulation by Apigenin

JAK/STAT signaling cascade is an evolutionary conserved signaling pathway involved in the regulation of plethora of cell processes, such as cell responses towards inflammation, migration, apoptosis, cell survival and cell growth [58]. Aberrant JAK-STAT signaling is a hallmark of various tumors [58]. In addition to above function, the JAK-STAT involvement in cancer cells growth and development has been thoroughly investigated in various animal models. The vital developmental processes, such as cell homeostasis, immune cell development, growth and stem cells development are regulated by the JAK-STAT signaling [59].

Apigenin has been investigated for its potential role in inhibiting this cell pathway to inhibit tumor cells growth and differentiation [60]. The mode of action of apigenin in modulating this evolutionary conserved cell pathway involves inhibition of JAK/SRC phosphorylation [61]. The repression of phosphorylation of key kinases prevents the activation of STAT3 which further prevents translocation of STAT dimmers to the nucleus and activation of key genes. STAT3 has been reported to regulate the expression of membrane metalloproteases (MMPs), TWIST1 and VEGF which are responsible for tumor invasion, migration and angiogenesis [23]. By preventing the STAT3 phosphorylation, apigenin prevents the activation of MMPs, TWIST1 and VEGF [23]. It has been evidenced from cell lines experiments conducted on SKBR3 and MDA-MB-453, HER2 positive cell lines that apigenin successfully reduces the expression of STAT3, STAT5 and JAK2 thus preventing tumor proliferation and growth. In addition to this, apigenin increased the expression of LMWPTP [62]. Briefly, LMWPTP is a negative regulator of STAT signaling pathway which prevents its active dimerization and translocation to the nucleus. There has been a study showing that apigenin is unable to modulate the expression of IL-6/STAT3 in SKOV3 and SKOV3/TR cell lines, suggesting that apigenin has limited effect on IL-6 expression [63].

### Wnt/β-Catenin signaling modulation by Apigenin

Wnt/β-catenin signaling plays a vital role in the development, differentiation, cell migration, organogenesis, neuronal patterning and tissue homeostasis [64]. Altered Wnt/β-catenin signaling has been involved in the progression and invasiveness of several tumors [65]. Apigenin modulates the expression of Wnt/β-catenin. Data has reported that the concentration of apigenin directly affects the Wnt/β-catenin expression [66]. Apigenin has also been reported to modulate the expression of downstream effectors of Wnt/β-catenin signaling, such as AXIN2, cyclin D1 and c-MYC [66]. Further investigations have revealed that apigenin has limited effect on LRP5 and Dishevelled (Dvl) and potentially targets the downstream molecules of Wnt/β-catenin signaling [66]. Apigenin prevents the accumulation and stabilization β-catenin in the cytoplasm in a dose-dependent manner and also hinders its translocation to the nucleus through repression of the PI3K/AkT/mTOR pathway [10]. Also apigenin overexpression prevents the metastasis and invasiveness of colorectal cancer through suppression of Wnt/β-catenin signaling, while promotes the expression of E-cadherin, hampering the translocation of β-catenin to the nucleus thus inhibiting the proto-oncogenes expression [67]. From these findings, it can be concluded that apigenin is a potential therapeutic option for the treatment of colorectal cancer.

### MAPK signaling modulation by Apigenin

Activated kinases, of the family mitogen activated protein kinase (MAPK), has three kinases that are considered pivotal for cell proliferation, differentiation and death. Categorically, the MAPK family is divided into 3 major classes, namely ERK/MAPK, c-JUN/SAPK and p38 kinase [68]. These kinases are necessary for cell growth and homeostasis. ERK protein family has several members that are exclusively involved in the modulation of MAPK-ERK pathway [69]. Over-expression of any ERK family member perturbs the normal functioning of both up and down stream targets of MAPK/ERK pathway [69]. In various types of cancer, the ERK signaling is activated by either mutation in kinases genes or in RAS and RAF, or by the activating mutations in the tyrosine kinase receptor. Apigenin has been stated to modulate the MAPK/ERK signaling. It has shown to suppress the proliferative ability of MAPK/ERK through modulation of the expression of AkT, ERK, DR4/DR5 and NF-kB [70, 71]. Apigenin administration promoted TRAIL-mediated apoptosis in human lung cancer cells [35]. In addition to this, apigenin has been reported to suppress growth, proliferation, metastasis and invasiveness of melanoma cells lines. Specifically, in both A375 and C8161 melanoma cell lines, the apigenin administration promoted the growth arrest via down regulation of the p-ERK1/2, p-AkT and p-mTOR [72]. In vitro and in vivo experimentation on colon cancer cell lines, HCT116 and DLD1, indicated that apigenin could trigger the AkT and ERK suppression under the influence of ABT-263 compound causing growth arrest and apoptosis [73]. Furthermore, apigenin has been reported to promote growth arrest and apoptosis in prostate cancer cells. Indeed, apigenin directly targets the IGF/IGFBP-3 and reduces its expression via modulation of p-AkT and ERK1/2 in a prostate cancer mice model [74]. Moreover, apigenin has been reported to be involved in the suppression of growth in human cutaneous melanoma cells. Apigenin suppresses the phosphorylation of ERK1/2 and FAK which in turn promotes poor integrin production and apoptosis [75]. On the contrary, apigenin has also been reported to elevate the expression of ERK in certain types of tumors. This has been well documented in the study conducted by the Shukla et al. [49], which demonstrated that apigenin treatment of LNCaP and PC-3 (prostate cancer cell lines) aggravated the levels of ERK1/2 and decreased the phosphorylation levels of p38 kinase. Conclusively these findings shed light on the fact that apigenin can trigger apoptosis via suppressing the expression of MAPK/ERK signaling pathway.

## Apigenin and miRNA interplay: a potential new avenue for cancer treatment

MicroRNAs (miRNAs) are small molecules having size between 10–25 bp and have gained a profound attention in the recent times because of their versatile role in genes transcription and translation [76]. In this section, the interplay between miRNAs and apigenin and how apigenin modulates the expression of the oncogenic and tumor suppressor miRNAs in cancer is discussed. Recently, it has come to limelight that apigenin in combination with certain miRNA mimics and miRNA inhibitors aided in the growth arrest and apoptosis of various cancer cells [60].

A combination of miRNA mimic and apigenin successfully triggered apoptosis in K-N-DZ and SK-N-BE2 cells. For example, in a study, the mice bearing the tumor were transfected with miR-138 mimic and apigenin for a period of 2 weeks in alternative days. After completion of the experiment, it was seen that the combine effect of miR-138 mimic and apigenin trigger tumor growth arrest in such tumor cells [77]. On the contrary, oncogenic miRNAs, such as *miR-423-5p* reversed the apoptotic effects of apigenin. Indeed, it has been reported that the glioma stem cell has elevated levels of miR-423-5p and its overexpression prevents growth arrest and apoptosis. However, miR-423-5p inhibitors suppressed the proliferative potential of glioma stem cells under the influence of apigenin while increased apoptosis [78].

Apigenin has been reported as a potentially sensitizer of cancer cells towards doxorubicin. It has come to light less lately that in BEL-7402 cells apigenin administration prevented chemo-resistance towards doxorubicin by modulating the miR-101 expression. Apigenin is responsible for upregulating the expression of miR-101 in BEL-7402. The miR-101 expression was seen to be downregulated in BEL-7402 cell, while the nuclear factor erythroid related factor (NRF2) was upregulated and conferred resistance towards doxorubicin. In the presence of apigenin, miR-101 directly targets NRF2 and downregulates its expression resulting in BEL-7402 cells sensitization towards doxorubicin [79]. It has been investigated overtime that rewiring key metabolic pathways could trigger chemo-resistance in various cancers. Mechanistic up-regulation of NRF2 by oncogenic KRAS triggers poor prognosis and confers chemo-resistance in patients with pancreatic cancer through altering glutamine metabolism. However, use of glutaminase inhibitors potentially sensitized pancreatic cancer cells towards gemcitabine and improved therapeutic efficacy [80]. From these findings it can be concluded that glutaminase inhibitors can be used as an alternate approach for the treatment of pancreatic cancer having KRAS mutations. It has been convincingly revealed that apigenin and miR-520b mimics sensitize BEL-7402/ADM cells towards doxorubicin in hepatocellular carcinoma. In the mice injected with miR-520b mimics there was a significant reduction in tumor aggression while tumor growth was reduced in mice treated with miR-520b mimics and apigenin [81]. Despite these evidences, the complex interactions between apigenin and miRNAs in cancer regulation have just been gleaned. However, how apigenin modulates the complex and broad spectrum of both oncogenic and tumor suppressor miRNAs still need to be explored. Therefore, in future studies a lot of emphasis should be given to the identification of miRNAs which are direct target of apigenin and how these approaches can be transformed into new therapeutic interventions for different cancers.

## The therapeutic potential of Apigenin in cancer treatment

To apigenin tremendous therapeutic and health benefits have been reported, with researchers being increasingly devoted to harvest its therapeutic potential [82]. So far, a number of patents have been filed demonstrating the anticancer potential of apigenin. Here, we have reviewed few of the studies which have been patented apigenin or that are in the process of being patented. A patent has been registered regarding a pharmaceutical composition that contains apigenin, curcumin and honokiol. This pharmaceutical composition has been found effective against lung cancer [83]. The pharmaceutical composition directly induced apoptosis in lung cancer cells thus preventing tumor growth, while facilitates chemoprevention. Briefly, it reduced the rate of glycolysis, ATP production, while downregulated ANT2 and suppressed PD-L1 [84]. Also, it has been reported that apigenin can modulate the epithelial to mesenchymal transition (EMT) [85]. A patent filed regarding the role of apigenin stated that it can both inhibit and invert the development of EMT process. Indeed, cells treated with apigenin present a decreased expression of mesenchymal marker and epithelial markers in a dose-dependent manner [86]. Similarly, another patent demonstrated that irradiated apigenin has more therapeutic potential in treating lung cancer cells as compared to the non-irradiated apigenin. Among the most prominent effects, irradiated apigenin triggers the ROS formation which in turn promotes apoptosis in lung cancer cell lines [87].

In addition, chyrophanol and apigenin mixed in a pharmaceutical composition exerted anticancer effects against choriocarcinoma cells. This composition prevented cell migration and increased the apoptosis rates in dose-dependent manner. In addition to this, the patented pharmaceutical composition when administrated with specific inhibitors of PI3k/Akt/mTOR and ERK signaling showed a tremendous efficacy. Moreover, the efficacy of chemotherapeutic drugs was increased by many folds when this formulation was used along with cisplatin and paclitaxel [88]. Several patents have been registered against apigenin as a potential anticancer agent. However, despite these efforts, there is still a need for more research input that can enhance the level of apigenin from bench to bedside.

## Apigenin nano-formulations for sustainable delivery

Wealth of information gleaned from previous decades in the field of nanotechnology has enabled researchers to design more target specific, efficient, cost-reductive nano-formulations able to eradicate a broad range of diseases [89]. Indeed, nano-based drug delivery systems are being highly adopted by worldwide researchers because of their precise targeting and limited cytotoxicity [90].

Natural compounds have gained a massive attention in the recent past because of their immense antioxidant, anti-proliferative, antifungal, antimicrobial and anti-pesticide properties [91]. Owing to these characteristics, several natural compounds-based drugs have been under clinical trials and few are even approved for treating different diseases. Nanoparticle-based approaches for the delivery of apigenin have revealed efficient and have tremendous potential as a therapeutic option for cancer. For instance, it has been reported that nanoparticles loaded with apigenin prevent hepato-cellular carcinoma proliferation in mice [92]. Apigenin linked gold nanoparticles (ap-AuNPs) have been reported to show anticancer activity in epidermoid squamous carcinoma cells A431. These linked nanoparticles showed limited cytotoxicity and apigenin itself acted as stabilizer. In addition to this, from the findings of chick chorioallantoic membrane assay (CAM) it was revealed that ap-AuNPs inhibited angiogenesis in A431 cells. Furthermore, it was found that ap-AuNPs triggered apoptosis and can be used as potential therapeutic option for skin cancer [93]. Nuclear factor E2-related factor 2 (Nrf2) confers chemo-resistance in non-small-cell lung cancer (NSCLC). The current therapeutic approach regarding the treatment of NSCLC involves docetaxel (DTX). However, presence of Nrf2 renders the chemotherapy approaches fruitless [92]. Apigenin has been reported as a potential inhibitor of Nrf2. A nano-formulation based on hyaluronic acid nano-structured lipid carriers (NLCs) carrying apigenin resulted in a decreased expression of Nrf2 in A549 NSCLC cells. Indeed, the administration of Ha-ApG-NLCs along with DTX resulted in an increased rate of apoptosis and decreased proliferation. Furthermore, Ha-ApG-NLCs administrated A549 cells when tested for real time-protein chain reaction (RT-PCR), a decreased expression of Nrf2, MRP2, HO-1 and Bcl-2 was observed. These findings reports that Ha-ApG-NLCs can be considered a suitable drug delivery platform and can reduce drug resistance in lung cancer [92].

One stumbling block that has hampered the emergence of apigenin as a drug candidate is its poor water solubility, limited bioavailability and non-specific distribution [94]. A recent study has devised a strategy for the efficient delivery of apigenin to the target site. An aptamer-conjugated apigenin loaded NPs (apt-ANPs) were designed and their anti-tumor and anti-proliferative abilities were checked on both in vitro and in vivo cancer models. These findings dictated the fact that Apt-ANPs were more target specific and increased the apigenin retention for colorectal carcinoma cells, thus can be used as a potential therapeutic delivery system for colorectal cancer because of their limited off-target cytotoxicity towards normal cells [95]. Another nano-formulation of apigenin has been reported as a potential anti-inflammatory and antioxidant agent for the treatment of acute lung injury and even lung cancer. Apigenin-loaded bovine serum albumin nanoparticles (BSA-Api-NPs) were prepared and their bioactive properties were measured using 2,2-diphenyl-1-picrylhydrazyl (DPPH^**⋅**^) free radical scavenging assay. The experimental findings revealed that BSA-Api-NPs is a potential novel delivery system against lung injury with good antioxidant abilities [96]. In addition, a recent study has shed light on the development of more efficient carrier/delivery system for apigenin. Mesoporous silica NPs are excellent nano-based drug delivery systems that can be implemented in the oral delivery of apigenin for clinical purposes [97]. Poly lactic-co-glycolide (PLGA) nanoparticles loaded with apigenin were found efficient in reducing Benzo[a]pyrene and ultraviolet B mediated skin cancer in mice model. This nano-formulation had an ameliorative effect on the treatment of cancer cell and can be used as a potential therapeutic option for the treatment of skin cancer [98].

Poly lactic-co-glycolide (PLGA) loaded with apigenin when administrated into A35 skin cancer cell line resulted in the active penetration of the nanoparticles into cells. Briefly, the encapsulated nanoparticles significantly enhanced apoptosis via degradation of mitochondria and breakdown of dsDNA. These findings suggest that PLGA loaded with apigenin can be used as a potential treatment option for skin melanomas [99]. A recent study conducted in the chronic myeloid leukemia cell line K562 to evaluate the efficacy of apigenin alone and loaded into chitosan-stearate nanogel ApG-SCS, revealed that the nano-gel loaded with apigenin induced apoptosis in a time-dependent manner and had more cytotoxic potential as compared to the normal apigenin not loaded in any nano-formulation [100]. Altogether, these emerging studies have indicated that apigenin nano-formulations can reduce chemo-resistance and the non-specific side effects caused by chemotherapeutic drugs. In addition to this, the use of nano-formulations for the treatment of different diseases requires considerably more research to bring it to clinical trial phases.

## Conclusion

Apigenin has significant anticancer potential both in vitro and in vivo [101]. This naturally-occurring bioflavonoid has been reported to have a low cytotoxicity that renders it highly specific and precise anticancer properties. Among others, apigenin modulates the key signaling molecules that participate in triggering cancer cells metastasis, proliferation and invasion. Nonetheless, the exact mechanism through which apigenin mediates cell growth inhibition via signaling cascades and molecular cross-talks are still unclear, so that to unveil these complex interactions, a plenty of research is still required. In spite of its vast anti-proliferative potential, one major stumbling block that has severely hampered the development of apigenin drug formulation is its low solubility in water and other organic solvents. Indeed, apigenin is relatively unstable in nature which makes it further difficult to develop sustainable drugs, but the transformation of apigenin into glycosidic and acylated forms can overcome solubility issues [82]. Owing to these features, new apigenin formulations are emerging, which easily dissolve in blood and can be easily excreted from urine [102]. However, there is still a debate regarding the high elimination and poor absorption of apigenin in the blood, with the limited pharmacokinetics and accumulation of apigenin in peripheral tissues being dictate its importance as a potent chemopreventive agent [102, 103]. In this way, nanoformulations have tremendously increased the bioavailability and efficacy of carrier drugs. Among others, the amalgamation of apigenin with nanoformulations has greatly raised its therapeutic efficacy and bioavailability, ultimately increasing its anti-tumor efficacy in various types of cancers [104]. Despite such experimental nanoformulations have proven efficient for apigenin drug delivery, the application of apigenin for humans still requires massive amount of evidence. Moreover, the pharmacokinetic profile of apigenin is still not available for humans, hampering the development of apigenin-based drugs. A plenty of research is required in the field of pharmacokinetics to establish the apigenin dosage for humans. As per the information available at the clinical trials.gov, there has been one study regarding the anti-tumor activity of apigenin in clinical trials (NCT00609310). So far this trial is reported to be suspended. The purpose of this trial was to evaluate effect of apigenin in hampering the tumor recurrence in colorectal cancer cells [105].

In short, considering the literature available so far, the use of apigenin in combinatorial therapies has been found effective, but still lacks substantial scientific evidences and certification from the regulatory authorities. In addition to this, the chemotherapeutic potential of apigenin is well documented but further evidences and research are required to validate apigenin as a potential chemotherapeutic agent. Moreover, the apigenin’ administration as chemotherapeutic agent must be done after obtaining substantial data from pre-clinical studies on humans, namely related to genetic predisposition, altered phenotypes and mutations that may trigger an unwanted response in humans. Taken together, apigenin has a plenty of benefits and can be implemented as a noble therapeutic agent if more and more studies are conducted on its ability to modulate cell signaling pathways, apoptosis and pathways involved in tumor progression.

## Data Availability

Yes.
